# Novel Anodic Catalyst Support for Direct Methanol Fuel Cell: Characterizations and Single-Cell Performances

**DOI:** 10.1186/s11671-018-2498-1

**Published:** 2018-04-03

**Authors:** N. Abdullah, S. K. Kamarudin, L. K. Shyuan

**Affiliations:** 1 Fuel Cell Institute, Universiti Kebangsaan Malaysia, 43600 Bangi, Selangor Malaysia; 2 Department of Chemical and Process Engineering, Universiti Kebangsaan Malaysia, 43600 Bangi, Selangor Malaysia

**Keywords:** Direct methanol fuel cell, Catalyst support, Titanium dioxide, Carbon nanofiber

## Abstract

This study introduces a novel titanium dioxide carbon nanofiber (TiO_2_-CNF) support for anodic catalyst in direct methanol fuel cell. The catalytic synthesis process involves several methods, namely the sol-gel, electrospinning, and deposition methods. The synthesized electrocatalyst is compared with other three electrocatalysts with different types of support. All of these electrocatalysts differ based on a number of physical and electrochemical characteristics. Experimental results show that the TiO_2_-CNF support gave the highest current density at 345.64 mA mg_catalyst_^−1^, which is equivalent to 5.54-fold that of carbon support while the power density is almost double that of the commercial electrocatalyst.

## Background

A direct methanol fuel cell (DMFC) is one of the most promising candidates for a renewable energy source. It is a power-generating system that produces electrical energy by converting the energy of a chemical liquid (methanol) fuel directly, without auxiliary devices. DMFCs are powered by their exciting possibilities in transportation and stationary application. Moreover, researchers also believe that this system is one of the most promising power sources for many mobile and portable applications, as well as a new alternative to rechargeable battery technology. DMFCs offer many advantages, including a smaller system size and weight. They are also clean energy carriers and lowering pollution. However, despite these advantages, they also face problems that hinder their commercialization. The most challenging problems are poor methanol electro-oxidation kinetics and low system performance. The overall cost needs to be reduced and problems such as methanol crossover, durability, stability, heat, and water management need to be improved for the DMFC to be successful [1–3].

The development of the DMFC began a decade ago by creating many solutions that utilized catalysts. One of the studies regarding the enhancement of DMFC performance analyzed different catalyst support structures, including carbon nanofiber (CNF), carbon nanotube (CNT), carbon nanowire (CNW), and other structure layers. The addition of new material into the catalyst, including a new hybrid catalyst creation, has also become a trend in DMFC evolution [4–6]. Despite this research, problems still exist, especially those related to low catalytic activity, stability, and conductivity for both electronic and protonic operation.

Platinum (Pt) is the best catalyst for both the hydrogen oxidation reaction (HOR) and the oxygen reduction reaction (ORR). Despite being effective in electrocatalyst applications [7–10], Pt is expensive, which requires more research to find a new catalyst. Currently, bimetallic platinum-ruthenium (PtRu) is the best catalyst for DMFC. The use of this catalyst reduced Pt loading, which reduced the electrocatalyst cost of DMFC with a standard ratio of 1:1. The role of Ru in this bimetallic catalyst is to remove the carbon monoxide (CO) from the active sites for the HOR which leads to surpass the CO poisoning of the catalyst [11, 12]. According to a study by Bock et al., PtRu showed superior catalytic activity in the DMFC, and it was clear that the catalytic performance strongly depended on the distribution of Pt and Ru sites at the atomic level [13]. However, the problem of low methanol oxidation cannot be solved using the PtRu catalyst, so further alteration of the catalyst must be undertaken to assist the fuel cell industry.

Metal oxides are the most prominent materials used to improve the electrocatalysis of DMFC. Titanium dioxide (TiO_2_), also known as titania, is an inorganic substance that is naturally stable, non-flammable, and highly resistant to corrosion. Titania is also not categorized as a hazardous substance by the United Nations (UN) Globally Harmonized System (GHS) of Classification and Labelling of Chemicals. In addition, the crystal structures of TiO_2_ are thermodynamically stable and help to create a composite material with good electrochemical and thermal stability [14]. The charge carriers from the anatase form become excited deeper in the bulk material, create more surface reactions, and incrementally improve the catalytic activity [15]. The electronic behavior of the material is enhanced when the interaction between the TiO_2_ metal oxide and the other material occurs. This enhancement can also improve the oxidation activity by lowering the CO oxidation potentials [6]. The use of TiO_2_ as a support for the metal catalyst affects the reaction kinetics and the reaction mechanism [16]. TiO_2_ has all of the previously listed benefits for many applications in various industries. Despite these benefits, the main problem precluding its widespread use for fuel cell applications is its low conductivity. However, to overcome this problem, high loadings of a Pt catalyst over TiO_2_ composited with an electric conducting material, such as N-doped carbon, and the employment of substoichiometric TiO_2_ are required [5].

This study reported the synthesis and characterization of titanium dioxide-carbon nanofiber (TiO_2_-CNF) deposited on a platinum-ruthenium (PtRu) catalyst for the DMFC application. The objective of the study was to synthesize the composite electrocatalyst with TiO_2_ in a nanofiber structure that can reduce the poisoning effect of the catalyst while enhancing the catalytic activity to improve the DMFC performance more than the commercial PtRu/C electrocatalyst. TiO_2_-CNF was prepared by electrospinning, followed by carbonization; finally, PtRu was deposited with an annotation of PtRu/TiO_2_-CNF. To characterize the prepared PtRu/TiO_2_-CNF composite electrocatalyst with different supports, X-ray diffraction (XRD), Brunauer-Emmett-Teller (BET), scanning electron microscope (SEM), and transmission electron microscope (TEM) were used. The performance of the electrocatalyst was evaluated by cyclic voltammetry (CV), electrochemical surface area (ECSA), Tafel analysis, chronoamperometry (CA), and DMFC single cell. All performance information was compared with several other supports, including C, CNF, and TiO_2_. Based on the experimental results, the effect of using the metal oxide as a support to improve the catalytic activity in the DMFC was discussed.

## Methods

### Materials

Titanium isopropoxide (TiPP, 97%) was obtained from Sigma-Aldrich Co., Ltd. Poly(vinyl acetate) (PVAc (Mw 500,000)), dimethylformamide (DMF (99.8%)), and acetic acid (99.7%) were received from Sigma-Aldrich Co., Ltd. Ethanol (99.8%) was purchased from R&M Chemical Reagents. These chemical reagents were used for nanofiber preparation. The deposition included a Pt precursor, H_2_PtCl_6_ (40% content), from Merck, Germany, and a Ru precursor, RuCl_3_ (45–55% content) and reducing agent, sodium borohydride (NaBH_4_, 96%), from Sigma-Aldrich Co., Ltd. The commercial catalyst support for C, CNF, and TiO_2_ nanopowder were obtained from Cabot Corporation, Cheap Tubes Inc., and Sigma-Aldrich Co., Ltd., respectively. The detailed properties for the catalyst support are tabulated in Table 1. All chemical reagents were used without further purification.

**Table 1:** Properties of commercial catalyst support

Catalyst support material	Product name	Purity (%)	Particle/diameter size (nm)
Carbon black (C)	Vulcan XC-72R	> 99	30
Carbon nanofiber (CNF)	VG-CNF	> 95	200–300
Titanium dioxide (TiO_2_)	Degussa P25	> 99.5	21

### Preparation of the TiO_2_-CNF

TiO_2_-CNF was synthesized using the sol-gel method and electrospinning technique. The PVAc (11.5 wt%) solution was prepared by dissolving the polymer with DMF for 1 h at a temperature of 60 °C and was continuously stirred overnight. 50 wt% of TiPP and a few drops of ethanol and acetic acid were mixed into the PVAc solution and stirred with a homogenizer until the mixture was homogenous. The mixed solution was fed from a syringe with a stainless steel needle for the electrospinning technique at a constant rate of 0.1 mL h^−1^, an applied voltage of 16 kV, and a distance of 18 cm between the tip and collector. The electrospun nanofiber was dried for 5 h at room temperature and continued to stabilize at 130 °C for 8 h. The fiber was carbonized using a tube furnace at 600 °C for 2 h in a nitrogen atmosphere. The calcined fiber then underwent a size controlling process using a mortar and pestle before further use in this study.

### Preparation of the Composite Electrocatalyst

All the electrocatalysts were synthesized using the deposition method by chemical reduction of NaBH_4_. The 20 wt% of PtRu with the atomic ratio 1:1 is loaded onto different catalyst supports, which are synthesized support, TiO_2_-CNF, and another three commercial supports, C, CNF and TiO_2_. A mixture of deionized water (DI water) and isopropyl alcohol (IPA) was added to the support material and sonicated for 30 min. The precursors were mixed into the support mixture and were continuously stirred for another 30 min until the solution was well mixed. The pH value of the solution was adjusted to 8 using a 1 M NaOH solution. Then, the temperature of the solution was increased to 80 °C. A 25-mL volume of a 0.2-M solution of NaBH_4_ was added dropwise into the solution and stirred for an additional hour. The mixture was cooled, filtered, and repeatedly washed with DI water. The electrocatalyst powder was dried for 3 h at 120 °C under a vacuum and crushed with a pestle and mortar to obtain a fine powder.

### Characterization of the Electrocatalyst

The X-ray diffraction (XRD) pattern and crystal structure for all of the electrocatalysts were investigated with an X-ray diffractometer (D8 Advance/Bruker AXS Germany) using powdered samples and operated at 40 kV and 20 mA. The surface area and pore size analysis using BET was handled by Micromeritics ASAP 2020 in the nitrogen adsorption/desorption isotherm condition at 77 K. A study of the surface morphology for the electrocatalyst-supported nanofibers and the prepared electrocatalyst was conducted using field emission scanning electron microscopy (FESEM (SUPRA 55 VP)). Mapping analysis was done to observe the distribution of the elements on a selected area in composite electrocatalyst. The detailed structure of the support and composite electrocatalyst was analyzed with high-resolution images obtained via transmission electron microscopy (TEM (Tecnai G2 F20 X-Twin)).

### Evaluation of the Electrochemical Measurement

Electrochemical measurements were evaluated by the Autolab electrochemical workstation. The methanol oxidation reaction (MOR) activity for the electrocatalyst was measured using the cyclic voltammetry (CV) of a three-electrode cell system. This system used a glassy carbon electrode (GCE, 3-mm diameter) as the working electrode and Pt and silver/silver chloride (Ag/AgCl) electrodes as the counter and reference electrodes, respectively, operated at room temperature. The working electrode must be cleaned with polish paper and alumina before being used. The preparation of the electrocatalyst ink for the working electrode was ultrasonically dispersed with 15 mg of the electrocatalyst in a mixture of 400 μL of DI water, 400 μL of IPA, and 125 μL of Nafion solution (5 wt%) for 30 min. A micropipette was used to transfer 2.5 μL of electrocatalyst ink onto a GCE. The working electrode was air-dried for 1 h at room temperature and then heated in an oven for 30 min at 80 °C. The working electrode was then ready for CV measurement. A solution of 0.5 M H_2_SO_4_ in 2 M methanol was prepared as an electrolyte. This electrolyte solution was bubbled with nitrogen gas (N_2_) for 20 min to achieve the oxygen-free content. The CV measurement was performed at a scan rate of 20 mV s^−1^, and the range of potentials was from 0 to 1.1 V vs. Ag/AgCl. The long-term performance of all electrocatalysts was assessed using chronoamperometry (CA) in the electrolyte solution at a potential of 0.5 V for 3600 s.

### MEA Fabrication

Membrane electrode assembly (MEA) consists of three main parts: membrane, anode, and cathode. Nafion 117 is selected as a membrane, and the membrane is treated to remove the impurities using hydrogen peroxide (H_2_O_2_) and DI water as applied in Hasran et al.’s [17] study. The treated membrane is stored in the beaker filled with DI water until it is ready to be used. Carbon cloth is used as the anode and cathode backing layer. This carbon cloth is treated with 5 wt% of polytetrafluoroethylene (PTFE) to make it waterproof. The carbon cloth is immersed into the PTFE solution and dried in the furnace for 30 min at 380 °C. The backing layer is coated with a gas diffusion layer of carbon, where the loading is 2 mg cm^−2^. The carbon is mixed with the IPA and Nafion dispersion D520 (Dupont). The carbon slurry is casted onto the carbon cloth and dried in the oven at 100 °C for 1 h. Then, the electrocatalyst layer is ready to be coated and synthesized by PtRu/TiO_2_-CNF and commercial electrocatalyst PtRu/C, used for the anode part, and Pt/C for cathode part. 2 mg cm^−2^ loading of electrocatalyst is added with IPA (1100 μL), DI water (300 μL), and Nafion dispersion (24 mg). The solution is dispersed in the homogenizer for 1 min and casted onto the carbon cloth. The anode and cathode are dried in the oven for 1 h at 100 °C. The anode and cathode are clamped together with membrane in the middle using hot press at the condition of 135 °C and 50 kPa for 3 min. The MEA is ready to be used in single-cell performance testing.

### Single-Cell Performance Testing

Performance testing of DMFC single cell was conducted in passive condition and room temperature. The MEA with a 4-cm^2^ active area is stated on the single cell, where the anode part is fixed at the methanol tank. Ten milliliters of 3 M methanol is fueled into the tank and tested using potentiostat/galvanostat (WonATech, Korea). The cell polarization curve is obtained for different electrocatalysts.

## Results and Discussion

### Structural Characterization

**Fig. 1:**
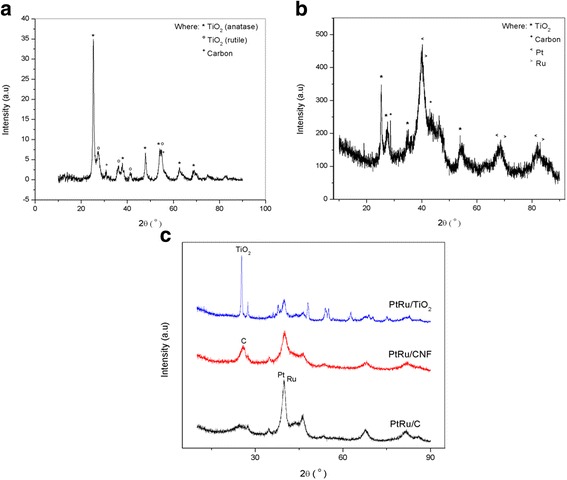
X-ray diffraction patterns. **a** TiO_2_-CNF, **b** PtRu/TiO_2_-CNF, and **c** compared electrocatalysts

The pattern and crystal structure of fabricated catalyst support, TiO_2_-CNF, synthesized electrocatalyst, PtRu/TiO_2_-CNF, and other electrocatalysts (PtRu/C, PtRu/CNF, and PtRu/TiO_2_) were investigated by XRD analysis. This analysis was completed using an X-ray diffractometer in the range of 5°–90° with 2*θ*, as shown in Fig. 1. The result for synthesized TiO_2_-CNF shows the existence of all materials, TiO_2_, and C. The diffraction peak of 25° (1 0 1) represents the TiO_2_ anatase structure, while peak at 27° (1 1 0) is the TiO_2_ rutile structure. These existing structures form in tetragonal structure (crystallographic structure for anatase and rutile) [18]. However, this sample had the anatase structure because the TiO_2_ structure changed from anatase to rutile while the sample was exposed to temperatures higher than 700 °C [19], whereas the temperature used in this research was only 600 °C.

The TiO_2_-CNF shows more diffraction peaks for TiO_2_ anatase at 38° (1 1 2), 48° (2 0 0), 55° (2 1 1), 63° (2 0 4), 69° (1 1 6), and 75° (2 1 5), while those of TiO_2_ rutile are 36° (1 0 1), 41° (1 1 1), and 54° (2 1 1). The carbon shows at the diffraction peaks of 31° (1 1 0) and 55° (2 1 1) in cubic structure. The XRD pattern for PtRu/TiO_2_-CNF electrocatalyst shows the diffraction peak for all the electrocatalysts involved, Pt, Ru, TiO_2_, and C. The peak for TiO_2_ and C is almost same with the TiO_2_-CNF sample, and the Pt and Ru stand out with another four peaks for each material, which is Pt at 39.7° (1 1 1), 46.2° (2 0 0), 67.5° (2 2 0), and 81.3° (3 1 1). The diffraction peaks for Ru are 40.7° (1 1 1), 47° (2 0 0), 69° (2 2 0), and 83.7° (3 1 1). Both of these metals come in the cubic structure. The synthesized electrocatalyst is compared with a few electrocatalysts supported with different catalyst supports, namely carbon black (PtRu/C), carbon nanofiber (PtRu/CNF), and titanium dioxide nanoparticle (PtRu/TiO_2_), and the diffraction pattern is figured out in Fig. 1c.

High Bragg angles were clearly visible, especially in the range of 25°–60° for the entire electrocatalyst sample. This showed that there was a bimetallic or alloy interaction that occurred in the catalyst [20]. A weak and broad intensity was observed for all of the electrocatalyst samples, which illustrates high dispersions in the prepared sample. The crystallite size was measured using the Debye-Scherrer equation [8]; crystallite size = 0.98*α*/*β*cos*θ*. Where *α* is the wavelength of the X-ray, *θ* is the angle at the peak, and *β* is the width of the peak at half-height. The value of the crystallite size was available via Eva software for analyzing the XRD results, and it was calculated using the Debye-Scherrer equation. The crystallite size for all samples is tabulated in Table 2. The crystallite size for PtRu was calculated as 4.64 to 9.84 nm, TiO_2_ ranged from 19 to 38.4 nm, and C was between 10.7 and 19.2 nm.

**Table 2:** PtRu, TiO_2_, and C crystallite sizes for all samples

Sample	PtRu crystallite size (nm)	TiO_2_ crystallite size (nm)	C crystallite size (nm)
TiO_2_-CNF	–	19	18.5
PtRu/TiO_2_-CNF	4.64	18.6	14.4
PtRu/C	9.84	–	10.7
PtRu/CNF	8.17	–	19.2
PtRu/TiO_2_	8.14	38.4	–

Surface area and porosity analysis of all electrocatalyst samples were analyzed using BET analysis. The nitrogen absorption/desorption isotherm is carried out at 77 K. The surface area, total pore volume, and average pore diameter of PtRu/TiO_2_-CNF, PtRu/C, PtRu/CNF, and PtRu/TiO_2_ electrocatalysts are listed in Table 3. The BET surface area for the nanofiber structure of metal oxide composite, PtRu/TiO_2_-CNF electrocatalyst, shows the lowest value with 50.59 m^2^/g, followed by PtRu/CNF, PtRu/TiO_2_, and PtRu/C electrocatalysts in ascending order. The results obtained in this study are close with the results of BET surface area conducted by other study [6], where PtRu/C electrocatalyst shows a much higher surface area compared to metal oxide composite electrocatalyst.

**Table 3:** BET analysis results for electrocatalyst PtRu/TiO_2_-CNF, PtRu/C, PtRu/CNF, and PtRu/TiO_2_

Electrocatalyst	*S*_BET_ (m^2^/g)	*V*_Total pore_ (cm^3^/g)	*V*_Micro_ (cm^3^/g)	*D*_Pore_ (nm)
PtRu/TiO_2_-CNF	50.59	0.227	0.0046	22.39
PtRu/C	143.92	0.734	0.0127	32.94
PtRu/CNF	55.46	0.370	0.0047	27.69
PtRu/TiO_2_	62.32	0.529	0.0016	32.10

The total pore volume, *V*_Total pore_, shows results in ascending order initiated by PtRu/TiO_2_-CNF < PtRu/CNF < PtRu/TiO_2_ < PtRu/C (0.227 < 0.370 < 0.529 < 0.734). The result pattern of the total pore volume and BET surface area is the same, indicating that an increase in the volume of the pore volume can increase the overall surface area of the electrocatalyst. Reduction of surface area and pore volume of PtRu/TiO_2_-CNF electrocatalyst is due to exposure and use of carbonization temperature up to 600 °C; meanwhile, other electrocatalyst do not undergo carbonization and exposure to high temperatures. This is due to the sintering effect, which subsequently leads to the growth of particles and crystallization [21].

**Fig. 2:**
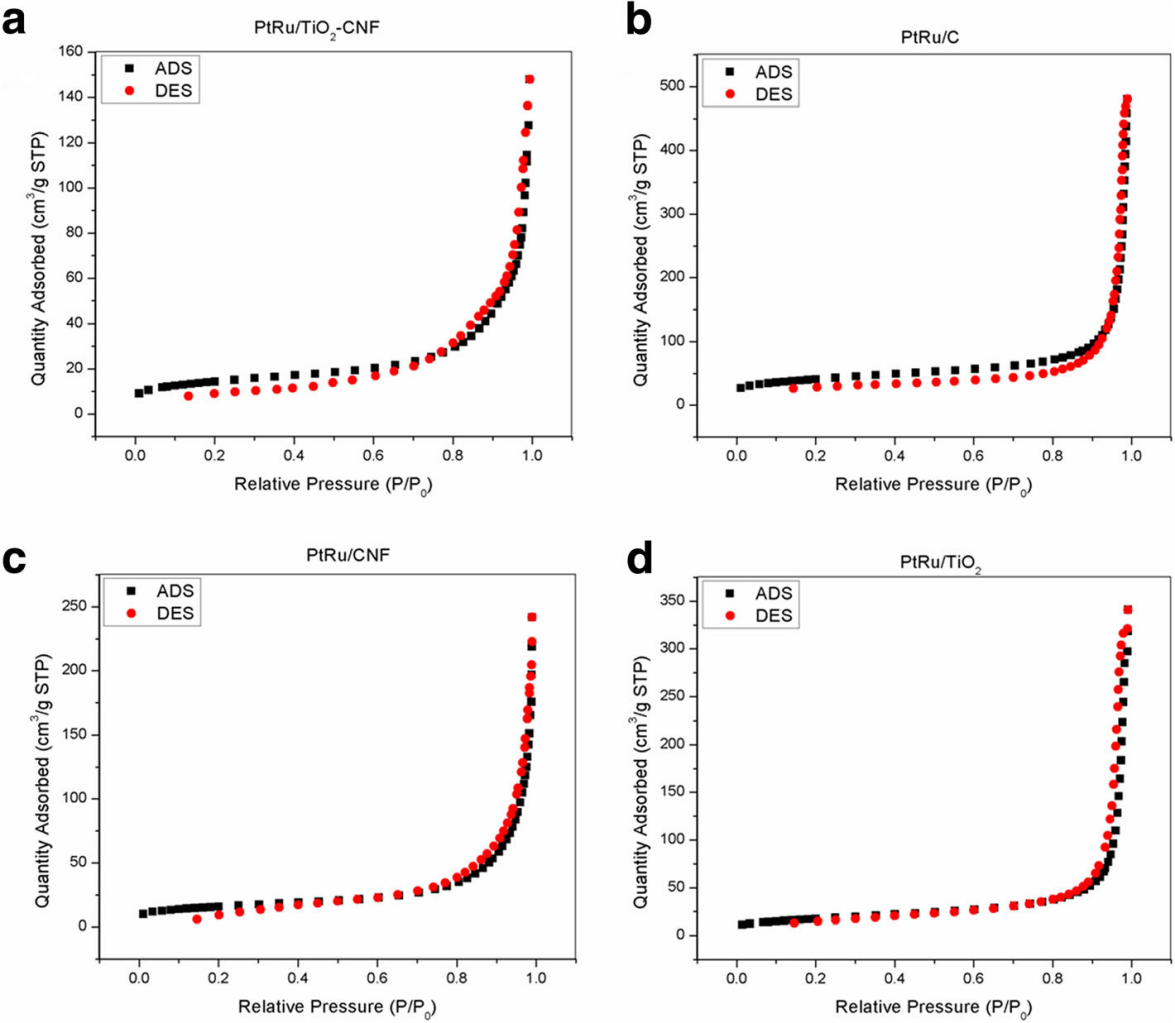
Nitrogen adsorption (ADS) and desorption (DES) isotherm at 77 K. **a** PtRu/TiO_2_-CNF, **b** PtRu/C, **c** PtRu/CNF, and **d** PtRu/TiO_2_

The nitrogen adsorption/desorption isotherm graphs at 77 K for all electrocatalysts are summarized in Fig. 2. The results show that the pores for all electrocatalyst samples highlight mesoporous properties, which have an average diameter within the range of 2–50 nm, which can largely be attributed to the large gap found in the electrocatalyst lattice. This type of electrocatalyst has the ability to increase the level of distribution and homogeneity of the immobilized catalyst, resulting in improving stability and catalytic activity [22].

The average pore diameter of the four electrocatalyst samples tested was between 22 and 33 nm, and the pore diameter of the PtRu/TiO_2_-CNF showed the lowest diameter compared to other electrocatalysts. Small diameter size illustrates the size of the particle as a whole. The small particle size has a high surface-to-volume ratio and potentially results in increasing surface reactivity and solubility and able to alter the toxicity profile of the substance. In addition, observation on the nitrogen adsorption/desorption isotherm graph appears flat at relatively low pressure (*P*/*P*_o_ ≤ 0.6), which may be due to the absorption of micropore in the sample. At a relatively high-pressure area (0.6 < *P*/*P*_o_ < 1.0), there is an increment in sample adsorption capability due to the adsorption of monolayer and/or multilayer nitrogen molecules in the meso-structure.

Figure 9 shows the SEM images for the nanofiber support, TiO_2_-CNF. The image illustrates that the nanofiber is produced smoothly without any agglomerates, beads, or connected nanofibers, which happens because of equivalent electrospinning parameters [23]. The distribution for the diameter size of TiO_2_-CNF is investigated by collecting 100 diameter measurements for this catalyst support and analyzed by the “Origin Software,” and the distribution diameter size is 136.73 ± 39.56 nm in the range of 90–170 nm.

The prepared electrocatalyst, PtRu/TiO_2_-CNF, also underwent SEM analysis, and the image is shown in Fig. 10. Figure 10a is the catalyst deposited on the support, PtRu/TiO_2_-CNF, after the milling process. The SEM image spotted the long fiber is covered with Pt and Ru nanoparticles. However, the image shows some agglomeration of Pt and Ru nanoparticles. To see the distribution of Pt and Ru, the mapping is shown in Fig. 10b for Pt and Fig. 10c for Ru. The results of the mapping illustrated that both metals were uniformly dispersed on the nanofiber; however, some agglomeration occurred for Pt due to an error during the deposition process. The agglomeration of nanoparticles was a reaction result because of overusing the NaOH solution during the pH adjustment for the deposition process [24].

The TEM images for the prepared catalyst support, TiO_2_-CNF, and electrocatalyst, PtRu/TiO_2_-CNF, are shown in Fig. 11. The TEM images of catalyst support Fig. 11a show that the TiO_2_ was homogenously dispersed in a 136-nm diameter of carbon nanofiber, due to the homogenous dispersion of polymer solution and TiO_2_ precursor during sol-gel method. Figure 11b shows the image of catalyst deposited on TiO_2_-CNF, where PtRu particles with a diameter of approximately 7 nm were deposited on the TiO_2_-CNF and exposed to the TiO_2_ surface. This connection and the exposure to TiO_2_ can produce a more active reaction spot during the performance. However, the PtRu particles agglomerated and were not homogenously distributed on the nanofiber surface.

### Electrochemical Characterization

**Fig. 3:**
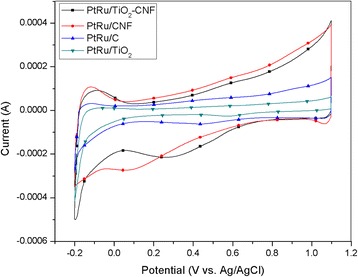
Cyclic voltammetry profiles of the different catalyst supports, PtRu/TiO_2_-CNF, PtRu/CNF, PtRu/C, and PtRu/TiO_2_ in 0.5 M H_2_SO_4_ solution at the scan rate of 20 mV s^−1^

The electrochemical characterization is applied to all catalysts, to see their potential and performance as anodic catalyst in DMFC. There are two main measurements in this section, which is cyclic voltammetry (CV), to measure electrocatalytic performance, and chronoamperometry (CA), to test long-term stability and durability of the samples. Figure 3 shows the CV profiles of all catalysts in 0.5 M H_2_SO_4_ solution in the potential range between − 0.2 and 1.2 V. The hydrogen adsorption/desorption region, in the range of − 0.2 to 0.1 V, is also indicated as the electrochemical active surface area (ECSA) is calculated. The ECSA is the estimation of PtRu nanoparticles surface area in the electrocatalyst [25]. The procedure involved a cycle of electrode current in the voltage range, where the charge transfer reactions are adsorption-limited at the activation sites. The total charge required for monolayer adsorption/desorption is used as reactive surface sites for ECSA [26]. The evaluated ECSA result is reported in Table 4. ECSA for the CV measurement was determined using the equation below:

$$ \mathrm{ECSA}\ \left({\mathrm{m}}^2{\mathrm{g}}_{\mathrm{Pt}}^{-1}\right)=\frac{Q}{\varGamma .{W}_{Pt}} $$

**Table 4:** Comparison of the current density results with the different catalyst supports

Catalyst	ECSA, [m^2^/g_PtRu_]	Peak potential, [V vs. Ag/AgCl]	Onset potential, [V vs. Ag/AgCl]	Peak current density, [mA/mg_PtRu_]	CO tolerance, *I*_f_/*I*_b_ ratio
PtRu/TiO_2_-CNF	10.4	0.639	0.305	345.64	4.73
PtRu/C	0.94	0.657	0.388	62.336	2.13
PtRu/CNF	8.4	0.542	0.315	186.29	15.4
PtRu/TiO_2_	0.76	0.686	0.571	16.03	2.51

where *Q* is the charge density or area under the graph ((C) of CV experiment), *Γ* (2.1 Cm_Pt_^−2^) is the constant for the charge required to reduce the proton monolayer on the Pt, and *W*_*Pt*_ is the Pt loading (g_Pt_) on the electrode. The ECSA calculation results show that the synthesized electrocatalyst, PtRu/TiO_2_-CNF, has the highest value of 10.4 m^2^ g_PtRu_^−1^, followed with PtRu/CNF (8.4 m^2^/g_PtRu_), PtRu/C (0.94 m^2^ g_PtRu_^−1^), and PtRu/TiO_2_ (0.76 m^2^ g_PtRu_^−1^). This happened because of several key factors. One of them is the crystallite size of PtRu, as mentioned in Table 2 from XRD analysis; the PtRu crystallite size for PtRu/TiO_2_-CNF is the smallest and shows a high ECSA value. The smallest crystallite size can elicit an increase in the catalyst and reaction surface area. The trend of crystallite size is followed with the trend of ECSA value for PtRu/CNF and PtRu/C. However, PtRu/TiO_2_ sample supposedly can produce higher ECSA value than PtRu/C, since the crystallite size is smaller, but the ECSA obtained is lower. This may happen due to the agglomeration of PtRu particle in the sample. This agglomeration can reduce the potential surface area to react and decrease the ECSA.

**Fig. 4:**
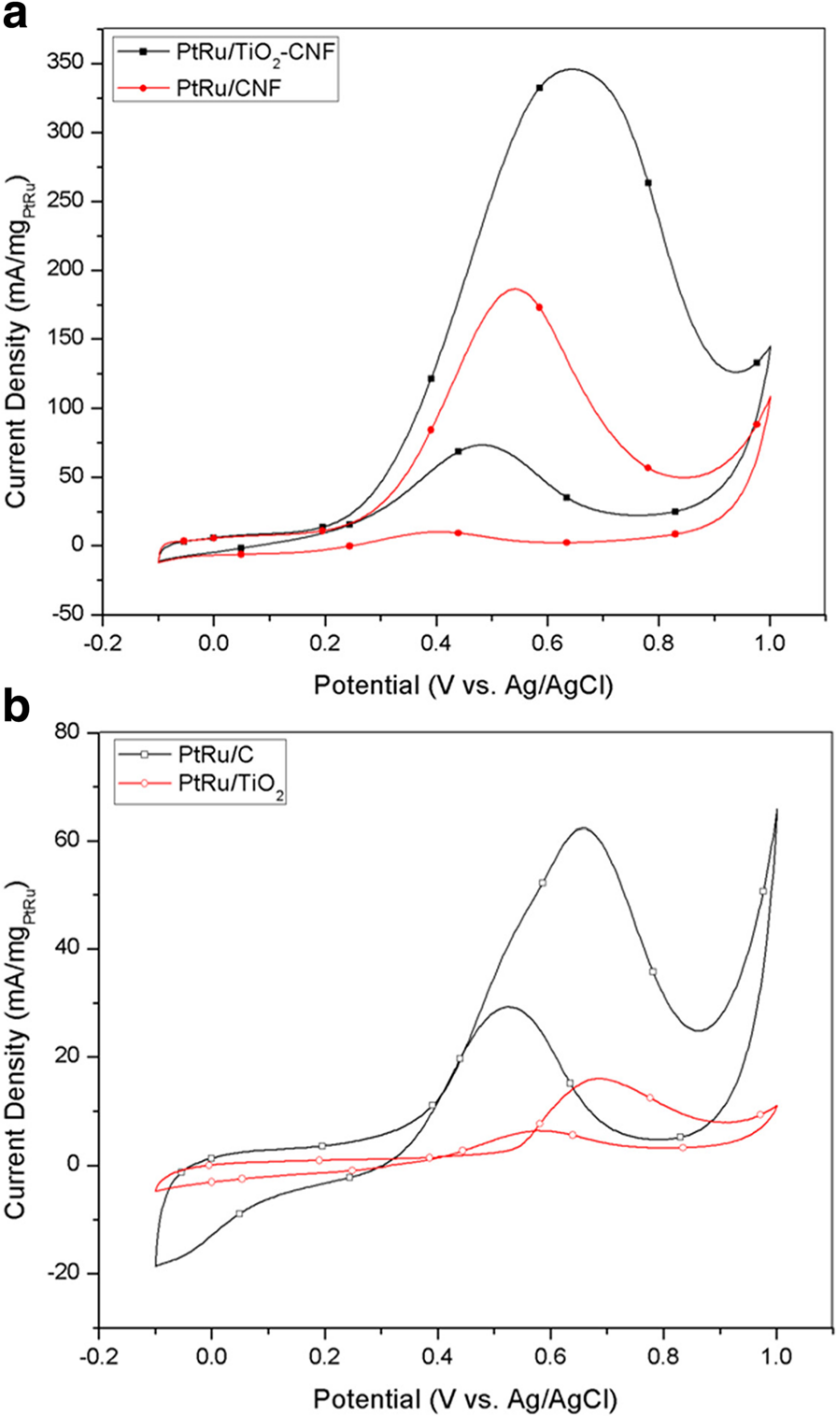
CV in 2 M methanol and 0.5 M H_2_SO_4_ at the scan rate of 20 mV s^−1^. **a** PtRu/TiO_2_-CNF and PtRu/CNF. **b** PtRu/C and PtRu/TiO_2_

The electrocatalytic performance of synthesized electrocatalyst and other electrocatalyst was analyzed with CV as illustrated in Fig. 4. The CV curve for the electrocatalysts, including PtRu/TiO_2_-CNF, PtRu/C, PtRu/CNF, and PtRu/TiO_2_, is measured in 2 M methanol with 0.5 M H_2_SO_4_ with saturated N_2_ gas at room temperature. The multiple curves are measured within the potential range of − 0.1 to 1.1 V vs. Ag/AgCl. Figure 4 shows that the peak current density in decreasing order was PtRu/TiO_2_-CNF > PtRu/CNF > PtRu/C > PtRu/TiO_2_. The peak current density of PtRu/TiO_2_-CNF for the MOR appeared to be approximately 0.639 V vs. Ag/AgCl. The peak current density and other CV values for all of the samples are reported in Table 4. The current density value for the PtRu/TiO_2_-CNF catalyst is 345.64 mA(mg_PtRu_)^−1^, which are 1.85 and 5.54 times higher than PtRu/CNF and commercial electrocatalyst, PtRu/C. This shows that the TiO_2_-CNF catalyst support was a better substitute for the carbon black catalyst support. This is because the nanofiber mixture, through the carbonization process, can increase the electro- and thermal conductivity of the catalyst [27].

Nanofiber structure in the composite electrocatalyst can increase the overall surface area and active reaction site on electrocatalyst surface area. Another advantage was the presence of high anatase TiO_2_ composition as resulted in XRD analysis. Higher electrocatalytic activity is acquired by anatase than rutile TiO_2_. The metal-support interaction also shows positive effect with higher peak current density, where the material combination between PtRu and TiO_2_-CNF exhibits a successful combination for electrocatalyst in DMFC. The second highest of peak current density with the value of 186.29 mA/mg_PtRu_ belongs to PtRu/CNF that is 2.99 times higher than commercial electrocatalyst, PtRu/C. This result is matched with the study by Zainoodin et al. [28] and Ito et al. [29]. The similarity of PtRu/TiO_2_-CNF and PtRu/CNF is the nanofiber catalyst support structure. The high peak current density for both samples demonstrates that the nanofiber can give an outstanding performance of methanol oxidation due to the capability of nanofiber to increase the electrocatalyst surface area and enhance catalytic activity. The performance for PtRu/C is much lower than that which resulted from the agglomeration of PtRu, where the ECSA value and crystallite size from XRD are featured. This situation reduces the potential of the electrocatalyst surface to be an active site and lowers the performance of electrocatalyst. The very low activity of PtRu/TiO_2_ was due to the nature of the TiO_2_ catalyst support having low electrical conductivity [4]. These results clearly show that the electro-conductive medium was essential for the catalyst systems for an electrochemical reaction [30].

The multiple CV curves in Fig. 4a, b show the reversed scan, and the small oxidation peak appears between 0.4 and 0.57 V vs. Ag/AgCl. Formation of incomplete oxidized carbonaceous species during the first oxidation peak resulted in the small oxidation on reversed scan also known as reversed oxidation peak [31]. This oxidation peak shows the tolerance of electrocatalyst towards the carbonaceous species by calculating the ratio of forward (*I*_f_) and reversed (*I*_b_) oxidation peak. The oxidation peak ratio called as CO tolerance is tabulated in Table 4. The result shows that both samples using nanofiber support, PtRu/TiO_2_-CNF, and PtRu/CNF have the highest electrocatalyst tolerance against carbonaceous species, which means these can lower the catalyst poisoning potential, with the ratio exceeding 4.7 respectively. This result shows that nanofiber structure and the combination of metal oxide in electrocatalyst can reduce the main problem faced by DMFC technology and have high potential to replace the commercial support used in this technology.

The synthesis electrocatalyst, PtRu/TiO_2_-CNF, is compared with other PtRu-based electrocatalyst, nanostructured catalyst support, and combination of metal oxide in electrocatalyst for DMFC technology and shown in Table 5. The result shows that the peak current density for PtRu/TiO_2_-CNF is the highest among other electrocatalysts. However, the high value of current density is obtained by using the nanostructure catalyst support and TiO_2_ as one of the side material in the composite electrocatalyst. Even though there are several different types of metal oxide used in the other study, the performance shows a gap with the TiO_2_-utilized electrocatalyst.

**Table 5:** Comparison of the performance results with the previous study

Authors	Type of catalyst	Peak potential, [V vs. RHE]	Peak current density (mA/mg_PtRu_)
This study	PtRu/TiO_2_-CNF	0.837	345.64
Nishanth et al. [31]	PtRu/TiO_2_-C	0.761	151.47
Lin et al. [32]	PtRu/CNT	0.857	66.69
Chen et al. [33]	PtRuWO_x_/C	0.913	56.02
Basri et al. [8]	PtRuNiFe/MWCNT	0.941	31
Yen et al. [34]	PtRu/MWCNT	0.913	326.4
Kolla & Smirnova [6]	TiO_2_-PtRu/C	0.697	324
Guo et al. [9]	PtRu_0.7_(CeO_2_)_0.3_/C	0.191	21.43

**Fig. 5:**
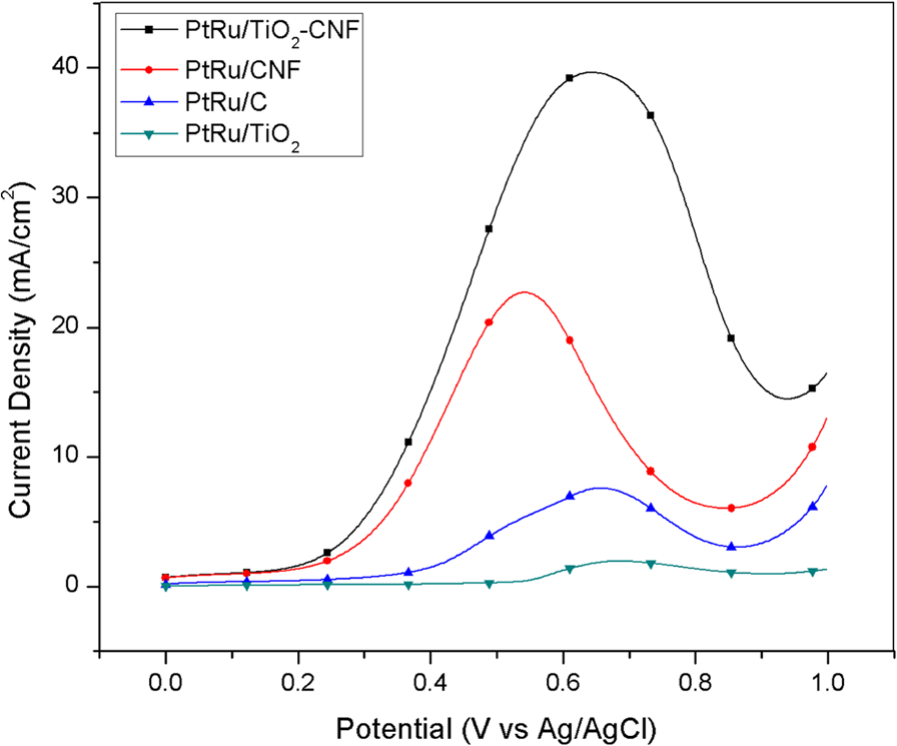
LSV in 2 M methanol and 0.5 M H_2_SO_4_ at the scan rate of 20 mV s^−1^ for all samples

Other than CV, linear sweep voltammetry (LSV) is one of the important electrochemical studies of electroactive substance. LSV is quite similar to CV, which measures the current response as a voltage function. Figure 5 shows the LSV plot for all the electrocatalysts that were measured in 2 M methanol and 0.5 M H_2_SO_4_ at the scan rate of 20 mVs^−1^ in the N_2_ gas environment. The result shows that the synthesized electrocatalyst, PtRu/TiO_2_-CNF, shows the highest current density that was calculated over the electrode surface area. The trend of the current density for LSV and CV is equalized. The LSV point shows the rising region between 0.5 and 0.7 V vs Ag/AgCl, and this region is known as a Tafel region that appeared when the electron transfer kinetics occur in the electrocatalyst surface [32]. The LSV data is extracted to present Tafel plot, where it relates the electrochemical reaction rate to the overpotential.

**Fig. 6:**
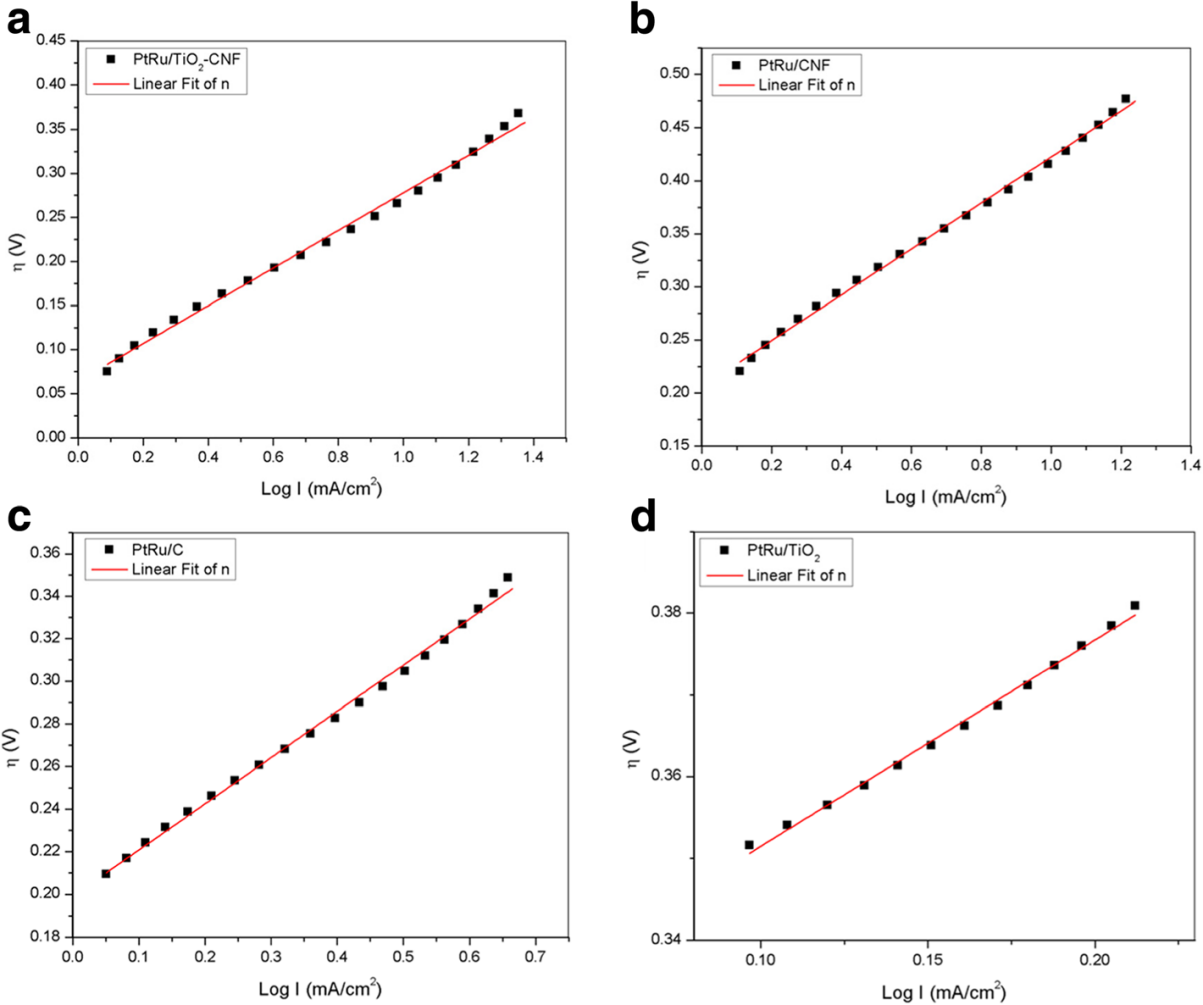
Tafel plot of the electrocatalyst **a** PtRu/TiO_2_-CNF, **b** PtRu/CNF, **c** PtRu/C, and **d** PtRu/TiO_2_

The Tafel plot of overpotential, *E*, against log *I* is presented in Fig. 6, and data extraction of the plot is tabulated in Table 6. This plot can provide and calculate the slope of anodic Tafel plot (*b*_a_) and ionic exchanging current density (*j*) from the slope and interception of the Tafel plot. Anodic Tafel slope, *b*_a_, for all the electrocatalyst has not much difference in value, while the ionic exchange current density gives a big gap between each electrocatalyst. The ionic exchange current density is also known as a catalytic activity explainer [33]. The *j* for all electrocatalysts shows the difference, where the highest value belongs to PtRu/TiO_2_-CNF with the value of 0.5012 mA cm^−2^. This result demonstrates that the synthesized electrocatalyst can produce the highest catalytic activity of bimetallic PtRu compared with other electrocatalyst. Even though the bimetallic composition for all the electrocatalysts is same, the synthesized electrocatalyst gets a greater help from the metal oxide in producing the highest active area for catalytic activity. PtRu/CNF and PtRu/C electrocatalysts have the same value, while PtRu/TiO_2_ has the lowest of ionic exchange current density with 0.112 and 0.046 mA cm^−2^, respectively.

**Table 6:** Data extraction from the Tafel plot

Type of catalyst	Tafel slope, *b*_a_ (mV/dec)	Ionic exchange current density, *j*_o_ (mA/cm^2^)	Standard deviation, *R*^2^
PtRu/TiO_2_-CNF	214	0.501	0.995
PtRu/CNF	217	0.112	0.997
PtRu/C	217	0.112	0.996
PtRu/TiO_2_	253	0.046	0.995

**Fig. 7:**
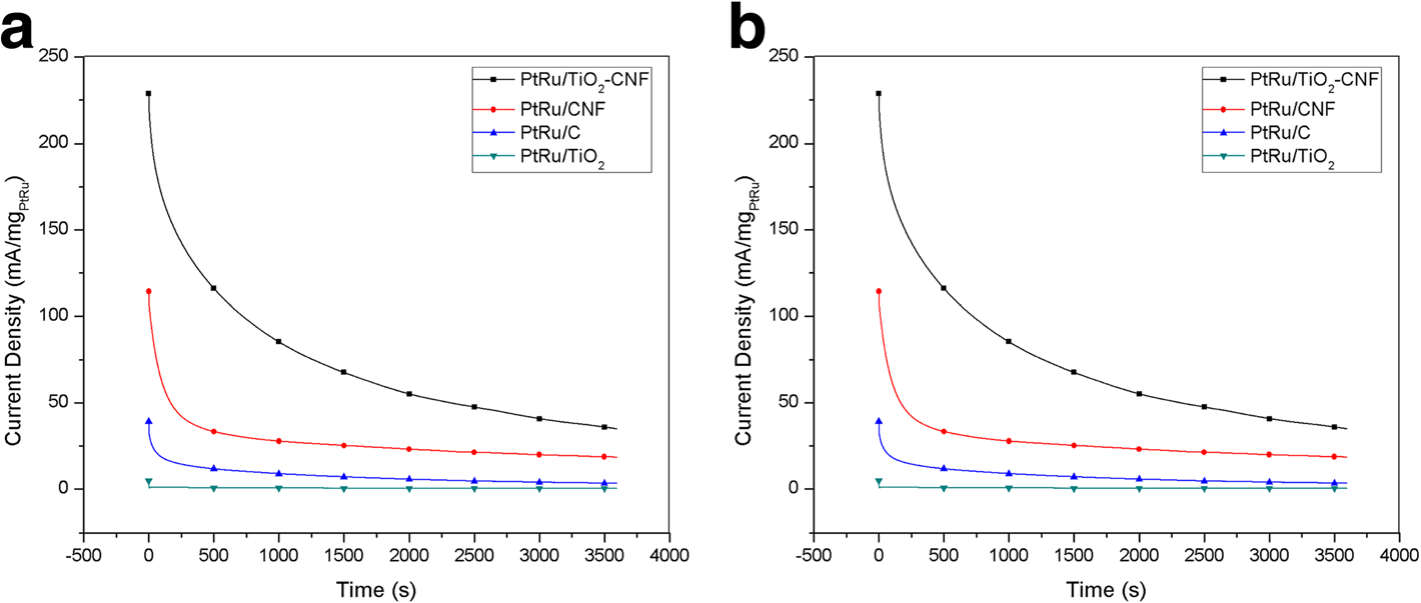
CA curve at potential of 0.5 V vs Ag/AgCl for PtRu/TiO_2_-CNF, PtRu/C, PtRu/CNF, and PtRu/TiO_2_ catalysts

The CA experiments were conducted to determine the stability and durability of the electrocatalyst for the long-term performance of MOR in a 2-M solution of methanol containing 0.5 M H_2_SO_4_ for 3600 s. Figure 7 shows the CA curve for the PtRu/TiO_2_-CNF, PtRu/C, PtRu/CNF, and PtRu/TiO_2_ electrocatalysts at a constant potential, 0.5 V. The current density of PtRu/TiO_2_ electrocatalysts shows the effect of a sharp drop at the start of the experiment, possibly due to the effect of poisoning by methanol oxidation mediation. The PtRu/TiO_2_-CNF, PtRu/CNF, and PtRu/C electrocatalysts showed a slight decline of approximately 5 and 3% in current density, respectively. After 3600 s, all of the electrocatalysts were stable, and the reducing current density ratios in increasing order are as follows: PtRu/CNF (6.16) < PtRu/TiO_2_-CNF (6.54) < PtRu/C (11.66) < PtRu/TiO_2_ (14.82). The PtRu/TiO_2_-CNF electrocatalyst showed the reducing current density ratio is slightly higher than PtRu/CNF, but this electrocatalyst reached the highest current density of all the electrocatalysts. This was due to good dispersion of the catalyst support and also to increased use of catalysis [6].

### DMFC Single-Cell Performance

**Fig. 8:**
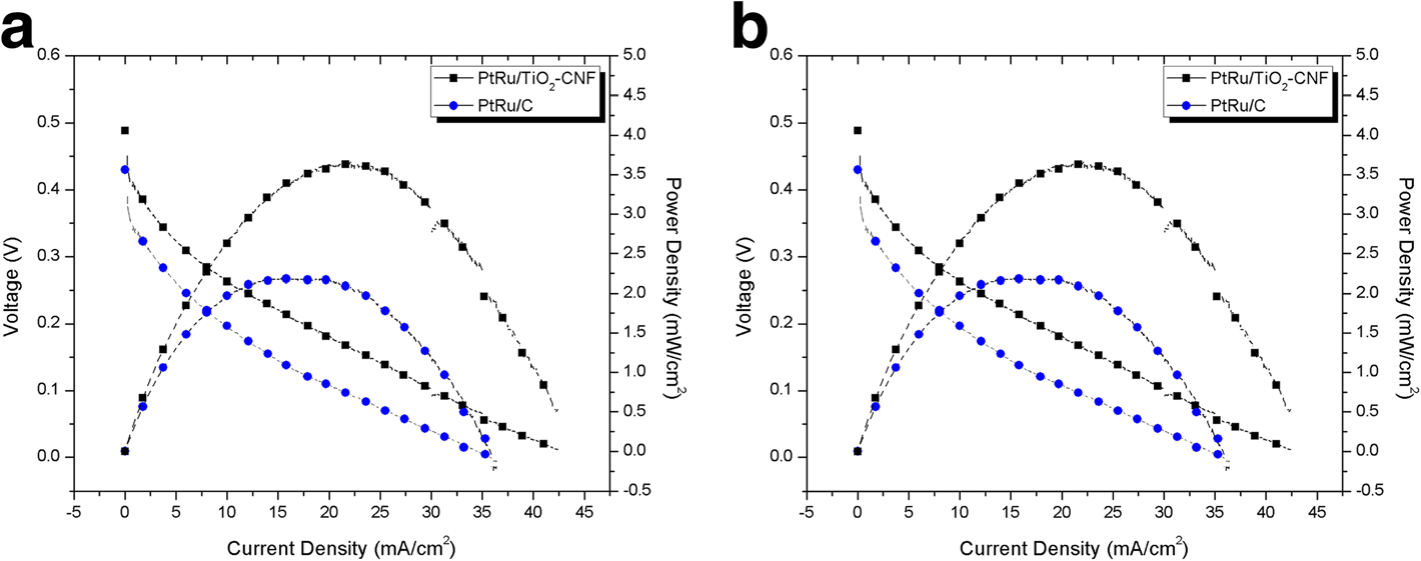
Current–voltage curve for PtRu/TiO_2_-CNF and PtRu/C in 3 M methanol with 2 mg cm^−2^ catalyst loading at room temperature

The synthesized electrocatalyst, PtRu/TiO_2_-CNF, with the highest electrochemical/half-cell performance was tested with single-cell performance. The performance is compared with commercial electrocatalyst, PtRu/C, using same composition, 20 wt% of PtRu. The 4-cm^2^ anode electrocatalyst layer clamped with cathode and membrane to be MEA, ready for single-cell performance using 3 M methanol of passive system. Figure 8 shows the current–voltage curve for PtRu/TiO_2_-CNF and PtRu/C. The PtRu/TiO_2_-CNF showed the highest performance compared to the commercial electrocatalyst, which is 1.66 times higher. The maximum power density for synthesized electrocatalyst was 3.8 mW cm^−2^, while PtRu/C was 2.2 mW cm^−2^.

**Fig. 9:**
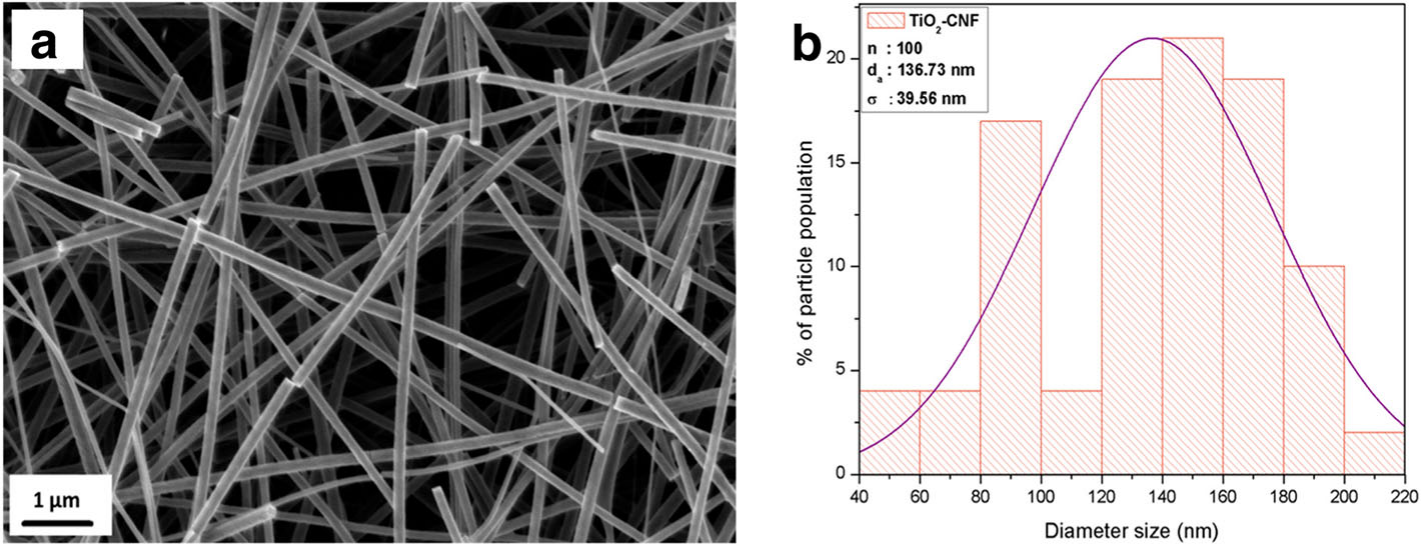
SEM images. **a** TiO_2_-CNF image (magnification × 10,000). **b** Distribution of diameter size for TiO_2_-CNF

**Fig. 10:**
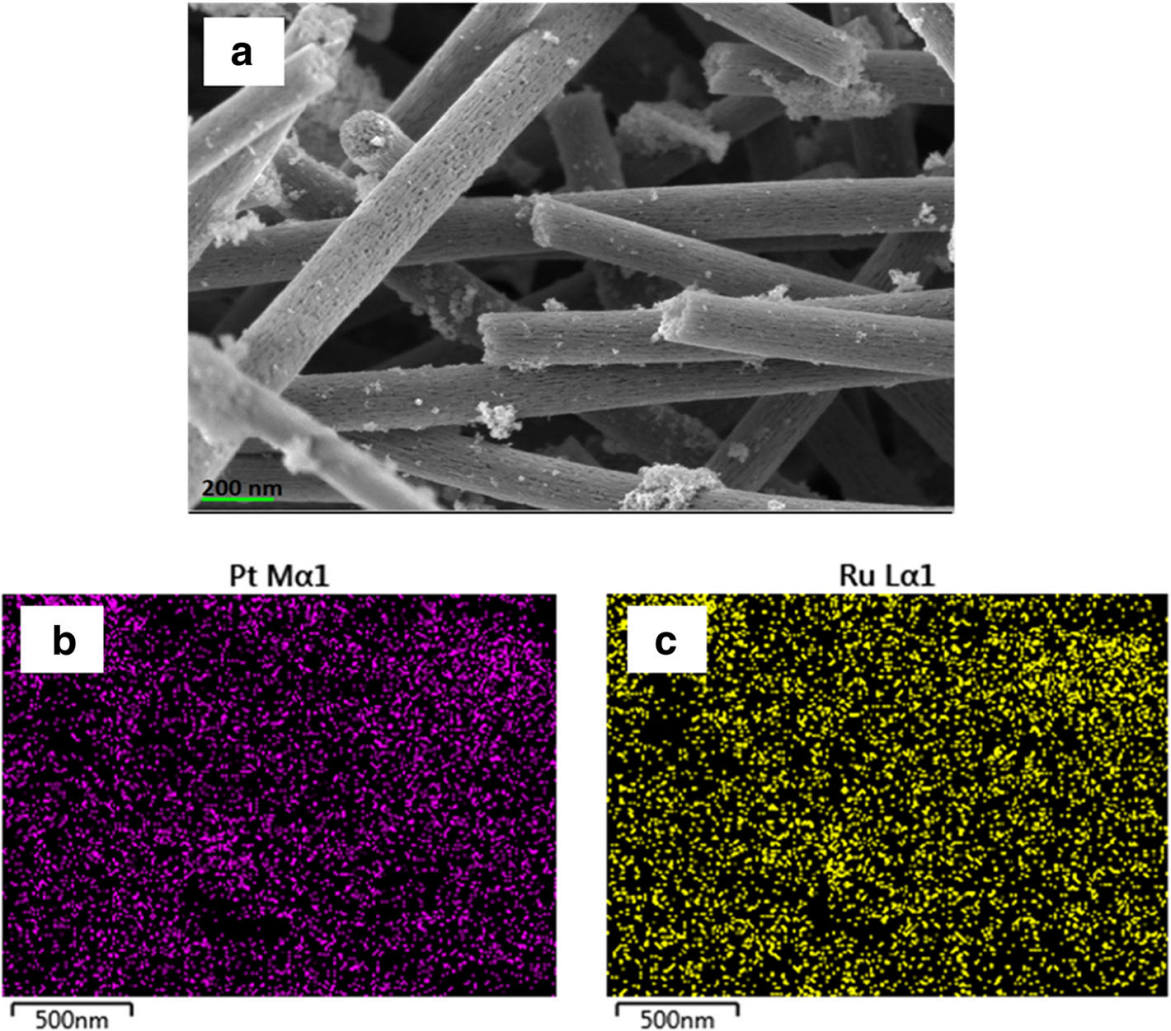
PtRu/TiO_2_-CNF catalyst after deposition and milling. **a** SEM images (magnification × 30,000), **b** mapping of Pt nanoparticles, and **c** mapping of Ru nanoparticles

**Fig. 11:**
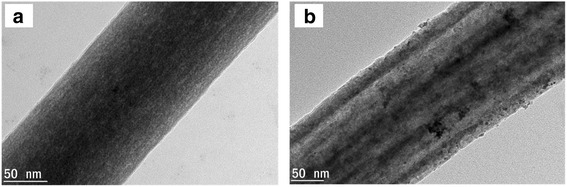
TEM images for prepared **a** TiO_2_-CNF catalyst support and **b** PtRu/TiO_2_-CNF electrocatalyst

The best PtRu/TiO_2_-CNF performance is confirmed by comparing this result with the previous study of commercial PtRu/C electrocatalyst, using the same catalyst loading in passive mode system that is tabulated in Table 7. The overall electrochemical and single-cell performance conclude that the combination of bimetallic catalyst, PtRu, and introduction of metal oxide nanofiber with carbon nanofiber have high potential to be replaced with PtRu/C in DMFC technology (Figs. 9, 10, and 11). By using the low composition of bimetallic catalyst and electrocatalyst loading, the synthesized electrocatalyst reveals the superior DMFC performance.

**Table 7:** Comparison of the single-cell performance results with the previous study

Study	Catalyst	Catalyst loading (mg/cm^2^)	Power density (mW/cm^2^)
This study	PtRu/TiO_2_-CNF	2	3.8
This study	PtRu/C	2	2.20
Shimizu et al. [35]	PtRu/C	2	3
Hashim et al. [36]	PtRu/C	2	1.7
Tang et al. [37]	PtRu/C	2	3.3

## Conclusions

The TiO_2_-CNF was synthesized using an electrospinning method and applied in the DMFC as a catalyst support for an anodic catalyst. The catalytic activity for the electrocatalyst was prepared for different catalyst supports including PtRu/TiO_2_-CNF, PtRu/C, PtRu/CNF, and PtRu/TiO_2_, which were compared with one another. The results showed that the prepared electrocatalyst, PtRu/TiO_2_-CNF, had the highest current density, which was 5.54 times higher than that of the commercial electrocatalyst, PtRu/C. The DMFC single-cell performance of PtRu/TiO_2_-CNF reveals the superior performance almost twice higher than that of PtRu/C. The highest catalytic activity was due to the nanofiber catalyst structure and the introduction of TiO_2_ as the catalyst support. The reaction with the metal support interface between the PtRu and TiO_2_-CNF catalysts helped to improve the properties of the catalyst layer. PtRu/TiO_2_-CNF is a promising candidate for support of the anode catalyst in DMFCs.

## Abbreviations

BET: Brunauer-Emmett-Teller
CA: Chronoamperometry
CNF: Carbon nanofiber
CNT: Carbon nanotube
CNW: Carbon nanowire
CV: Cyclic voltammetry
DI: Deionized
DMF: Dimethylformamide
DMFC: Direct methanol fuel cell
ECSA: Electrochemical surface area
GCE: Glassy carbon electrode
GHS: Globally Harmonized System
HOR: Hydrogen oxidation reaction
IPA: Isopropyl alcohol
LSV: Linear sweep voltammetry
MEA: Membrane electrode assembly
MOR: Methanol oxidation reaction
ORR: Oxygen reduction reaction
PTFE: Polytetrafluoroethylene
SEM: Scanning electron microscope
TEM: Transmission electron microscope
UN: United Nations
XRD: X-ray diffraction
